# Perception of the importance of continuing professional development among pharmacists in a middle east country: A cross-sectional study

**DOI:** 10.1371/journal.pone.0283984

**Published:** 2023-04-14

**Authors:** Rula M. Darwish, Khawla Ammar, Asma Rumman, Saad M. Jaddoua

**Affiliations:** 1 Department of Pharmaceutics and Pharmaceutical Technology, School of Pharmacy, The University of Jordan, Amman, Jordan; 2 Survey Unit, King Hussein Cancer Centre, Amman, Jordan; 3 Department of Pharmacy, King Hussein Cancer Centre, Amman, Jordan; University of Turin, ITALY

## Abstract

**Background:**

The role of pharmacists has recently expanded, necessitating enhanced competencies. This requires pharmacists’ participation in continuing education initiatives. This study aims to explore attitudes, motivations, opportunities, and challenges of pharmacists in a country in the middle east, towards continuous professional development.

**Methods:**

A cross-sectional observational close-ended questions study was conducted in Jordan between September and October 2021, and enrolled 309 pharmacists, the tool was developed by the research team and experts in the field to evaluate the continuous professional development perception among pharmacists. The research was approved by the Ethics and Research Committee in an area hospital and a University.

**Results:**

The majority of participants had confidence that continuous professional development prepares pharmacists for practical development, believed that it enhances the status of the profession with other health care professionals as well as with the public, and felt confident that it meets their needs (> 98%). Most of the participants agreed that job restrictions (91%) and lack of time (83%) were the major barriers to participation in continuous professional development. The motivation was positively correlated with attitudes (R = 0.551, P < .001). However, barriers were not significantly correlated with either attitudes or motivations.

**Conclusion:**

Our findings emphasize the positive attitude of pharmacists towards continuous professional development. Identified barriers to continuous professional development participation included job constraints and lack of time. The study highlights the need for policies and procedures that address these issues before the implementation of mandatory continuous professional development programs for pharmacists.

## Introduction

Most professions, particularly those involved with health care, are becoming increasingly more aware of the importance of continuous professional development (CPD) [1]. CPD refers to the idea that learning continues throughout the professional career to maintain appropriate experience in the practice of the health care practitioner, keeping up-to-date with continuing education and professional competency and undertaking appropriate development and training opportunities that are relevant to the practice [2–5]. The role of the pharmacist has changed over the years to become more patient centered than product centered. Thus, in order to accomplish effective pharmacy practices, well-established measures and standards must be followed and continuously maintained and this is accomplished by CPD [6]. CPD for pharmacists is a lifetime learning process that includes any education that helps them improve their skills, whether it is workplace-based, distance learning, or electronic learning [7, 8]. This ongoing process is based on the four-stage Kolb learning cycle of identification, planning, implementation/action, and evaluation [9]. In the Middle East, mandatory CPD requirements were adopted in the UAE in 2014, Qatar in 2016, and Lebanon in 2014 while other countries, such as Egypt, are in the process of assessing the level of readiness and acceptance of CPD in the healthcare sector [10–13]. In Jordan, there were no educational requirements of licensure prior to 2018. Starting from 2018, license renewal would need the completion of CPD or education as approved by the High Health Council and Jordan Medical Council (JMC) [14]. Pharmacists practicing in Jordan are required to document 50 credit hours in five years, with an average of 10 credits of CPD activity annually.

Jordan Pharmaceutical Association structured some local guidelines in 2021 to control the different activities related to CPD to ensure quality and assess the development of competencies [15]. These guidelines would help in regulating and evaluating the process of training. As a result, each pharmacist has to submit his/her records for review and monitoring. Failure to meet these standards could lead to license suspension. For pharmacists in many countries, the transition to a CPD strategy and the usage of a learning portfolio for documentation and reflection is still relatively new. There is scarce published evidence on the influence of such changes on pharmacists’ perspectives and attitudes toward CPD [16, 17]. A study done in Northern Ireland in 2001 on pharmacists’ attitudes toward continuing education highlighted lack of time, compensation, and lack of understanding of CPD as barriers to participation [18]. Another study conducted on a limited number of English community pharmacists found that they required further help because they were not completely engaged in CPD [19]. Hospital pharmacists indicated higher levels of confidence in the CPD process, whereas community pharmacists were regarded as the pharmacy sector that required the most assistance in terms of the ability to participate [5]. The first large-scale study analyzing the function of CPD in the pharmacy sector was conducted in the United States, aiming to determine whether a structured educational intervention would help pharmacists use a CPD strategy [20]. To the best of our knowledge, there has been no published information in Jordan about pharmacists’ perspectives and attitudes toward CPD. Thus, the primary goal of this study is to evaluate the perspectives and attitudes of pharmacists in Jordan, a middle Eastern Country, towards mandatory CPD hours and to identify sectors where pharmacists’ enthusiasm for CPD is high, as well as major hurdles to participation.

## Methods

### Study design and setting

This is a cross-sectional observational close-ended questions study targeting practicing Jordanian pharmacists. Pharmacists working on Jordanian premises were included in the study, whereas pharmacists living outside Jordan were excluded. The study was conducted between September and October 2021. An introductory paragraph outlining the purpose of the study was posted along with the survey. The survey link was posted on pharmacist social media pages and sent to WhatsApp groups through focal points in targeted settings, in addition the link was also posted on pharmacists institution pages. Participants had the study information on the cover page and were informed that participation was voluntary, anonymous and they could withdraw at any point. Completing the survey was considered as consent from the participants.

### Population

The study targeted around 10,000 pharmacists with estimated response rate of 25%, thus, at least 285 completed surveys were needed to achieve a 95% confidence interval. Three hundred and nine participants completed the survey with completion rate of 99%. The sample size was estimated based on Roasoft [18, 19, 21–24]. For quality control purposes, participants were able to complete the survey only once to limit redundant answers, missing data was reported to be less than 4% in some of the questions. The questionnaire was prepared following a thorough examination of the literature and based on previous similar studies conducted in different countries, and sentences were carefully structured taking in consideration societal needs [17–19, 21–25]. Two clinical pharmacists finally reviewed the tool for content and face validity. A preliminary test was also conducted on a convenient sample of 10 pharmacists to address any ambiguity in the questions and to determine whether the data would provide reliable information. Additional changes were taken into consideration after the pilot. The data collected during the pilot phase of the study were not included in the final analysis.

### Instrument

The questionnaire consisted of closed-ended questions and were divided into four parts including demographic information of the participants, attitudes of pharmacists to different elements of CPD (Identification, Planning, and Implementation), barriers to participation in CPD activities, and motivation towards (CPD). The statements were written in clear language and answer options were in four points Likert scale (strongly disagree, disagree, agree and strongly agree). The data collection lasted for 2 months, September and October 2021.

### Statistical method and data analysis

The Statistical Package for the Social Sciences (SPSS 28) was used to analyze the data. For each variable, descriptive statistics were computed. Frequencies and percentages were reported, chi square and Fisher’s exact tests were used to assess the association of the categorical variables, and p ≤ 0.05 was considered significant. Mean and standard deviation were used for reporting continuous variables. One-way Anova test was used to assess associations. Correlation between attitudes, barriers, and motivation was assessed by Pearson correlation, with P value ≤ 0.05 being considered significant.

Statements were grouped as; attitudes, 5 statements, barriers 9 statements, and motivations 7 statements. The answer options were given scores from 4 to 1 descending, scores for each domain were summed to show the total score, higher scores indicate higher positive/agreement level. The total score of each dimension was computed by summing the scores for different statements in each dimension, higher scores indicating a higher agreement level. The attitude response scores ranged from 6 to 20, with a higher score indicating a better/more positive attitude for CPD. Five statements were used to assess attitude including; “CPD is essential to improve the professional practice”, “CPD enhances status of the profession with other health care professionals”, “CPD enhances status of the profession with the public”, “I want to engage more on CPD”, and “CPD should be mandatory”. Barriers were assessed by the following nine statements: “Accessibility of learning activities”, “Job constraints”, “Lack of time”, “Cost of participation”, “Lack of relevant learning opportunities”, “Uninteresting subjects or topics”, “Lack of quality learning’, “Family constraints”, and “Subjects are too specialized”. The total barrier scores ranged between 16 and 36, with higher score indicating higher number of barriers. Finally, motivation statements included “Confidence that CPD meets the pharmacist’s needs”, “Confidence that CPD is preparing pharmacist for practice development”, “Sufficient time to achieve pharmacist CPD goals”, “Sufficient resources”, “Challenges in the job that motivate pharmacists to achieve my CPD goals”, “Live conferences with colleagues motivate pharmacist to achieve my CPD goals” and “sufficient enthusiasm to achieve my CPD goals”. The score range was 7–23.

## Results

The comparison of the demographic data between those who were aware of CPD and those who were not are shown in Table 1. Results showed that participants of age more than 50 were more aware about CPD (p < 0.001). Awareness about CPD was more among those with higher educational degrees (masters and PhD; p < 0.001). Participants working in the academia field were more aware about CPD, and this was consistent with the educational level. Awareness increased with higher income (p = 0.013). Pharmacy owners were more aware about CPD than employees (p = 0.02). The results also showed that the higher the experience, the more the awareness about CPD (> 15 years; p < 0.001). Pharmacists in professional organizations had higher levels of awareness of CPD (p = 0.001).

**Table 1:** Comparison of demographic data between participants who are aware about CPD and those who are not.

		Aware of CPD	Not aware of CPD	Total	
		Frequency, %	Frequency, %		P
**Age**	18–30	71, 49.7%	72, 50.3%	143, 46.4%	<0.001
	31–50	91, 70.5%	38, 29.5%	129, 41.9%	
	Older than 50	35, 97.2%	1, 2.8%	36, 11.7%	
**Gender**	Male	63, 63%	37, 37%	100, 32.5%	0.808
	Female	134, 64.4%	74, 35.6%	208, 67.5%	
**Pharmacy degree**	Pharmacy (BSc)	90, 56.6%	69, 43.4%	159, 51.8%	0.000
	Pharmacy D	39, 58.2%	28, 41.8%	67, 21.8%	
	Pharmacy MSc	22, 78.6%	6, 21.4%	28, 9.1%	
	Pharmacy PhD	45, 84.9%	8, 15.1%	53, 17.3%	
**Sector**	Private	137, 59.6%	93, 40.4%	230, 74.7%	0.011
	Public	49, 74.2%	17, 25.8%	66, 21.4%	
	Other	11, 91.7%	1, 8.3%	12, 3.9%	
**Working place**	Academia	51, 91.1%	5, 8.9%	56, 17.9%	<0.001
	Registration	4, 66.7%	2, 33.3%	6, 2%	
	Community pharmacy	28, 82.4%	6, 17.6%	34, 11.1%	
	Chain pharmacy	4, 44.4%	5, 55.6%	9, 2.9%	
	Hospital pharmacy	58, 64.4%	32, 35.6%	90, 29.3%	
	Industry	8, 47.1%	9, 52.9%	17, 5.5%	
	Sales	22, 34.9%	41, 65.1%	63, 20.5%	
	Other	22, 66.7%	11, 33.3%	33, 10.7%	
**Monthly income**	200–399	15, 46.9%	17, 53.1%	32, 10.5%	0.013
	400–599	35, 68.6%	16, 31.4%	51, 16.7%	
	600–799	42, 54.5%	35, 45.5%	77, 25.2%	
	> 800	104, 71.2%	42, 28.8%	146, 47.7%	
**Employment status**	Owner	23, 85.2%	4, 14.8%	27, 8.8%	0.020
	Employee	174, 61.9%	107, 38.1%	281, 91.2%	
**Experience**	Less than a year	19, 52.8%	17, 47.2%	36, 11.7%	<0.001
	1–4	40, 47.1%	45, 52.9%	85, 27.6%	
	5–9	41, 64.1%	23, 35.9%	64, 20.8%	
	10–14	30, 85.7%	5, 14.3%	35, 11.4%	
	> 15	67, 76.1%	21, 23.9%	88, 28.6%	
**Membership to any professional organization**	Yes	156, 69.3%	69, 30.7%	225, 73.1%	0.001
	No	41, 49.4%	42, 50.6%	83, 26.9%	

Table 2 showed that 98% (N = 303) participants felt that CPD is essential to improve their professional practice as well as enhancing the status of the profession among other healthcare providers and the public. There was no statistical difference between participants who were aware of CPD and those who were not in regard to the importance of CPD for professional practice and enhancing the status of the profession among other healthcare professionals and the public. There was a statistical difference in terms of their willingness to be more engaged in CPD activities in favor of those who were aware of the CPD concept (97.4%, N = 191 vs 92.7%, N = 101). Around 82.4% (N = 252) of the participants believed that CPD should be mandatory. There was no significant difference in this regard between both groups, those who were aware and those who were not aware of CPD (82.7% vs 81.7%, p = 0.811). In relation to motivation including having confidence that CPD meets the needs of pharmacists and is preparing them for practical development, the majority of participants agreed (around 94.7%, N = 287) with no statistical difference between the two groups (p = 0.896). Most participants (87.2%) agreed that they have sufficient resources (computer access, internet access, and conferences) to achieve their CPD goals There was no association between those who agreed that they have sufficient resources and those that know about CPD. There was significant difference (p < 0.001) regarding agreement on the lack of relevant learning opportunities between those who were aware about CPD and those who were not (65.5% and 85.2%, respectively). Again, the results showed that those who were not aware of CPD (67.6%) were more in agreement with the lack of interesting subjects or topics. Only 35.1% of those who were aware of CPD agreed that subjects for CPD were too specialized in comparison to 55% of those who were not aware of CPD. As for family constraints, there was significant difference between the two groups with more agreeing to this statement than those who were not aware of the concept of the CPD (55%). However, results in Table 3 show no statistical difference in demographic characteristics between the two groups in relation to family constraints. Results in the supplementary file show significant correlation between motivation and attitudes. However, barriers are not significantly correlated with either attitudes or motivations. Table 4 shows the comparison of mean scores for motivation, attitudes, and barriers based on gender, age groups, and awareness about CPD. Significant difference was found in attitude towards CPD in participants older than 50 years old (p = 0.042). Awareness about CPD was significantly associated with scores; participants who were aware of CPD were more motivated, had a better attitude, and had less barriers towards CPD than those who were not (p = 0.01, p < 0.001, and p = 0.041 respectively).

**Table 2:** Comparison of percentages and frequencies of agree/disagree to different statements for attitudes, barriers, and motivation between those participants who are aware about CPD and those who are not.

		Aware of CPD		Not aware of CPD		P
		Agree	Disagree	Agree	Disagree	
**Attitudes**						
**12**	CPD is essential to improve the professional practice	196	1	107	3	0.133
		99.5%	0.5%	97.3%	2.7%	
**13**	CPD enhances status of the profession with other health care professionals	195	2	107	3	0.256
		99%	1%	97.3%	2.7%	
**14**	CPD enhances status of the profession with the public	190	6	104	5	0.493
		96.9%	3.1%	95.4%	4.6%	
**15**	I want to engage more with CPD	191	5	101	8	0.047
		97.4%	2.6%	92.7%	7.3%	
**16**	CPD should be mandatory	163	34	89	20	0.811
		82.7%	17.3%	81.7%	18.3%	
**Barriers**						
**17**	Accessibility of learning activities (distance)	157	38	90	19	0.760
		80.5%	19.5%	82.6%	17.4%	
**18**	Job constraints	173	20	101	7	0.299
		89.6%	10.4%	93.5%	6.5%	
**19**	Lack of time	156	38	96	13	0.109
		80.4%	19.6%	88.1%	11.9%	
**20**	Cost of participation	149	43	92	16	0.131
		77.6%	22.4%	85.2%	14.8%	
**21**	Lack of relevant learning opportunities	127	67	92	16	< 0.001
		65.5%	34.5%	85.2%	14.8%	
**22**	Uninteresting subjects or topics	100	88	73	35	0.020
		53.2%	46.8%	67.6%	32.4%	
**23**	Lack of quality learning activities	129	63	79	30	0.366
		67.2%	32.8%	72.5%	27.5%	
**24**	Family constraints (restrictions)	80	113	60	49	0.030
		41.5%	58.5%	55%	45%	
**25**	Subjects/ topics are too specialized	68	126	60	49	< .001
		35.1%	64.9%	55%	45%	
**Motivations**						
**26**	Confidence that CPD meets the pharmacist’s needs	184	9	102	7	0.512
		95.3%	4.7%	93.6%	6.4%	
**27**	Confidence that CPD is preparing a pharmacist for practical development	184	10	103	6	0.896
		94.8%	5.2%	94.5%	5.5%	
**28**	Sufficient time to achieve pharmacist CPD goals	163	32	91	17	1
		83.6%	16.4%	84.3%	15.7%	
**29**	Sufficient resources (computer, internet)	186	27	94	14	1
		86.2%	13.8%	87%	13%	
**30**	Sufficient enthusiasm to achieve CPD goals	171	25	91	17	0.470
		87.2%	12.8%	84.3%	15.7%	
					5	
**31**	Challenges in the job that motivate the pharmacist to achieve CPD goals	184	11	96	12	0.085
		94.4%	5.6%	88.9%	11.1%	
**32**	Live conferences with colleagues motivate pharmacist to achieve CPD goals	117	18	98	10	0.993
		90.8%	9.2%	90.7%	9.3%	

Note: p values were calculated to assess the level of association, and p ≤ 0.05 was considered significant.

**Table 3:** Comparison based on family constraints for the whole sample.

		Family constraints		P
		Agree	Disagree	
**Age Group**	**18–30**	57, 40.4%	84, 59.6%	0.078
	**31–50**	68, 54%	58, 46%	
	**Older than 50**	15, 42.9%	20, 57.1%	
**Gender**	**Male**	45, 45.9%	53, 54.1%	1
	**Female**	95, 46.6%	109, 53.4%	
**Pharmacy degree**	**Pharmacy (BSc)**	67, 42.9%	89, 57.1%	0.209
	**Pharmacy D**	30, 46.2%	35, 53.8%	
	**Pharmacy MSc**	12, 42.9%	16, 57.1%	
	**Pharmacy PhD**	31, 59.6%	21, 40.4%	
**Sector**	**Private**	102, 45.1%	124, 54.9%	0.344
	**Public**	34, 53.1%	30, 46.9%	
	**Other**	4, 33.3%	8, 66.7%	
**Working place**	**Academia**	32, 57.1%	24, 42.9%	0.376
	**Registration**	4, 66.7%	2, 33.3%	
	**Community pharmacy**	13, 39.4%	20, 60.6%	
	**Chain pharmacy**	5, 62.5%	3, 37.5%	
	**Hospital pharmacy**	37, 42%	51, 58%	
	**Industry**	5, 31.3%	11, 68%	
	**Sales**	30, 48.4%	32, 51.6%	
	**Other**	14, 42.4%	19, 57.6%	
**Income**	**200–399**	18, 58.1%	13, 41.9%	0.344
	**400–599**	20, 39.2%	31, 60.8%	
	**600–799**	32, 43.2%	42, 56.8%	
	**More than 800**	70, 48.6%	74, 51.4%	
**Employment status**	**Owner**	9, 34.6%	17, 65.4%	0.225
	**Employee**	131, 47.5%	145, 52.5%	
**Experience**	**Less than a year**	10, 28.6%	25, 71.4%	0.157
	**1–4**	41, 49.4%	42, 50.6%	
	**5–9**	27, 42.2%	37, 57.8%	
	**10–14**	17, 50%	17, 50%	
	**More than 15 years**	45, 52.3%	41, 47.7%	
**Membership to any professional organization, like Jordan Pharmaceutical Association**	**Yes**	102, 46.2%	119, 53.8%	1
	**No**	38, 46.9%	43, 53.1%	

**Table 4:** Comparison of means for motivation, attitudes, and barriers based on gender, age groups, and awareness about CPD.

			N	Mean	Std. Deviation	Minimum	Maximum	P
**Gender**	**Motivations**	**Male**	96	28.6979	2.55516	19.00	35.00	0.210
		**Female**	204	29.1422	2.99168	21.00	35.00	
		**Total**	300	29.0000	2.86251	19.00	35.00	
	**Attitudes**	**Male**	99	21.5960	2.35571	11.00	25.00	0.074
		**Female**	206	22.0922	2.21221	17.00	25.00	
		**Total**	305	21.9311	2.26788	11.00	25.00	
	**Barriers**	**Male**	93	19.7742	3.49263	9.00	28.00	0.253
		**Female**	198	19.2727	3.47527	9.00	29.00	
		**Total**	291	19.4330	3.48269	9.00	29.00	
**Age group**	**Motivations**	**18–30**	139	28.8849	2.73485	21.00	35.00	0.126
		**31–50**	127	28.8740	2.99468	19.00	35.00	
		**Older than 50**	34	29.9412	2.77370	27.00	35.00	
		**Total**	300	29.0000	2.86251	19.00	35.00	
	**Attitudes**	**18–30**	143	21.8252	2.10404	17.00	25.00	0.042
		**31–50**	128	21.8047	2.46250	11.00	25.00	
		**Older than 50**	34	22.8529	2.00200	18.00	25.00	
		**Total**	305	21.9311	2.26788	11.00	25.00	
	**Barriers**	**18–30**	140	19.7214	3.40560	9.00	29.00	0.085
		**31–50**	117	18.8974	3.65396	9.00	29.00	
		**Older than 50**	34	20.0882	2.99866	12.00	27.00	
		**Total**	291	19.4330	3.48269	9.00	29.00	
**Awareness about CPD**	**Motivations**	**Yes**	191	29.3298	2.90029	21.00	35.00	0.010
		**No**	108	28.4444	2.71491	19.00	35.00	
		**Total**	299	29.0100	2.86202	19.00	35.00	
	**Attitudes**	**Yes**	195	22.4872	2.11855	16.00	25.00	< 0.001
		**No**	109	20.9817	2.17299	11.00	25.00	
		**Total**	304	21.9474	2.25383	11.00	25.00	
	**Barriers**	**Yes**	184	19.7554	3.55134	9.00	29.00	0.041
		**No**	106	18.8868	3.31898	9.00	28.00	
		**Total**	290	19.4379	3.48769	9.00	29.00	

## Discussion

This was the first prospective, cross-sectional study on the attitudes, motivation, and barriers for pharmacists in an attempt to introduce CPD as a structured approach to learning and professional development for them in Jordan. It is essential to assess pharmacists’ attitudes, motivation, and barriers in order to build a complete CPD program that would improve their competencies. Literature shows that attitudes and motivation are critical components in CPD participation [26]. Our results showed that participants generally have confidence that CPD meets pharmacists’ needs and prepares them for practical development. These outcomes are in agreement with those of a study conducted on Scottish pharmacists’ views and attitudes towards CPD [27]. However, it contradicts findings of other studies which state that “general medical practitioners’ insights into their own educational needs were also poor” [28]. Knowledge about the concept of CPD has no effect on participants’ perceptions about the importance of CPD to meet the pharmacists’ needs and that it would prepare pharmacists for practical development. This is an interesting finding in that those who were aware and those who were not aware of the concept of CPD welcome the idea and that would mean that introducing a CPD program is well received by all. There was a significant difference in the belief of the participants in relation to the lack of relevant learning opportunities and uninteresting subjects or topics. This might be due to the differences in perception of learning needs between members of the study group, who received specific education in the area of CPD and are capable of selecting activities that helped them meet their needs and those who didn’t; this was in agreement with other studies [28]. That may indicate the need to develop the knowledge and skills of pharmacists to enable them to identify their own learning needs proactively and to make these sources accessible. In addition, the findings may suggest that when a CPD program is proposed, the needs and the topics that are of interest to the pharmacists must be investigated. The participants of the CPD program need to be involved and this will in turn motivate them more. In general, our study showed that motivation and attitudes were positively correlated. These findings are consistent with the findings of a study conducted on the attitudes and preferences of Texan pharmacists regarding continuing pharmacy education, which revealed that approximately 83% of the participants found that currently available CE programs met their educational needs [29]. Most participants (274, 91%) indicated job constraints as one of the barriers with no statistical difference between those who are aware of CPD concept and the others. This is in agreement with the findings of Aldosari et al. (2020) on attitudes and perceptions of pharmacists in Jordan [30]. Another study in Lebanon on 525 pharmacists showed that the job restrictions associated with CPD and lack of personal time and motivation were the major barriers [24]. Motivation was found to be significantly correlated with pharmacist attitudes and significantly negatively correlated with barriers in that study. In the current study, however, there was significant correlation between motivation and attitudes but barriers are not significantly correlated with either attitudes or motivation. Researchers in different countries reported similar barriers to the current study that might play a role in discouraging pharmacists from participation in CPD activities including time constraints, lack of motivation, inaccessibility, and quality of learning material and the methods used to deliver CPD [31, 32]. Thus, job constraints should be further explored when structuring CPD program. Other major barrier according to our study is family constraints. The results show no significant difference in this regard among participants according to age group, gender, pharmacy degree, sector, working place, income, employment status, or experience. This contradicts another study in Ethiopia which showed that family constraints were more cited as a barrier to CPD engagement by the females (X2 = 4.546, p = 0.033), more educated (X2 = 6.002, P = 0.014), and pharmacy owners (X2 = 4.785, p = 0.029) than their counter groups [30]. Probably, it is just the anticipation from those participants that since CPD needs time and commitment, this can affect family time. This could suggest that pharmacists could not undertake CPD outside their work hours because of family and social commitments. Thus, it is recommended to keep this point in mind when designing CPD programs. Further studies are required to explore how to overcome the reported barriers and provide more feasible and relevant continuing education to pharmacists. There was a significant correlation between pharmacists’ awareness of CPD and age, academic degree, working place, monthly income, employment status, experience, and membership to any professional organization according to our results. This may be due to the fact that higher qualifications and association with professional organizations in addition to financial status reinforces the value of CPD. In that regard, pharmacists who attain higher education are more committed to becoming lifelong learners than their colleagues with lower qualifications [31]. Our study showed significant associations between attitudes and age, and this is in agreement with studies in the literature [24]. However, other studies indicated that age and experience where negatively influencing CPD involvement because some participants felt too old to learn new material and others felt they were experienced enough to not engage in CPD [32]. This study explored knowledge, attitudes, and motivation towards CPD among pharmacists, who are important members of the multidisciplinary healthcare providers’ team. Owing to their accessibility to the public, they could have a positive impact on patients’ health outcomes. The study addressed a very important issue that is the concept of CPD among pharmacists, who are accessible healthcare providers. The timing of the study is of value since most authorities all over the world realized the importance of CPD among healthcare workers to improve patients’ outcomes. Barriers have been identified, thus it would be beneficial to be addressed in any policy or action plan regarding CPD. The only limitation, the research team believe in is the low response rate- just as reported in online surveys in general- as a result, more than minimum required sample size was included in the analysis in order to be as representative as feasible, though the research team tried to control factors like targeting the population, make the survey short, and make it easy to answer by checking the questions flow, layout and answers options before starting the data collection.

## Conclusion

To increase pharmacist participation in CPD activities, it is important to first assess pharmacist attitudes regarding CPD and then identify the barriers that prevent pharmacists from participating in CPD activities. This is the first study in Jordan to investigate pharmacists’ perceptions toward CPD. Almost all the participants have favorable attitudes toward CPD. Despite having positive opinions regarding CPD, various barriers have been identified, with the most commonly stated barriers being job constraints, lack of time, cost of participation, and lack of relevant learning opportunities. Policymakers must be aware of such obstacles before generalizing CPD programs in order to have effective solutions that break down barriers and inspire pharmacists to better pursue their CPD. Guidelines are required to set up a system for obligatory CPD for pharmacists. Incorporating CPD points in annual achievement reports or making them a condition for pharmacist license renewal is essential. This would keep Jordan in line with other countries that have defined and mandatory CPD systems. Given the findings of this study, it may be useful to target young pharmacists with less experience and lesser qualifications.

## Supporting information

S1 Data (XLSX)
